# Crystal structure of 5-*tert*-but­yl-10,15,20-tri­phenyl­porphyrin

**DOI:** 10.1107/S2056989016000025

**Published:** 2016-01-09

**Authors:** Keith J. Flanagan, Ebrahim Mohamed Mothi, Lisa Kötzner, Mathias O. Senge

**Affiliations:** a School of Chemistry, SFI Tetrapyrrole Laboratory, Trinity Biomedical Sciences Institute, 152-160 Pearse Street, Trinity College Dublin, The University of Dublin, Dublin 2, Ireland; b Centre for Scientific and Applied Research, PSN College of Engineering and Technology, Melathediyoor, Tirunelveli 627 152, India

**Keywords:** crystal structure, unsymmetrical porphyrins, free base porphyrin, non-planar porphyrins.

## Abstract

In the title free base porphyrin, C_42_H_34_N_4_, the neighbouring N⋯N distances in the center of the ring vary from 2.818 (8) to 2.998 (8) Å and the phenyl rings are tilted from the 24-atom mean plane at angles varying between 62.42 (2)–71.63 (2)°. The NH groups are involved in intra­molecular bifurcated N—H⋯(N,N) hydrogen bonds. The C_a_—C_m_—C_a_ angles vary slightly for the phenyl rings, between 124.19 (18)–126.17 (18)°. The largest deviation from the mean plane of the 24-atom macrocycle is associated with the *meso* carbon at the substituted *tert*-butyl position, which is displaced from the mean plane by 0.44 (2) Å. The free base porphyrin is characterized by a significant degree of *ruffle*d (*B*_1*u*_) distortion with contributions from *dome*d (*A*_2*u*_) and *wave* [*E_g_*(*y*) and *E_g_*(*x*)] modes. In the crystal, mol­ecules are linked by a number of weak C—H⋯π inter­actions, forming a three-dimensional framework. The structure was refined as a two-component inversion twin.

## Abstract 2

In the title free base porphyrin, the neighbouring N⋯N distances in the center of the ring vary from 2.818 (8) to 2.998 (8) Å and the phenyl rings are tilted from the 24-atom mean plane at angles varying between 62.42 (2) to 71.63 (2)°. The free base porphyrin is characterized by a significant degree of *ruffled* (*B*_1*u*_) distortion with contributions from *domed* (*A*_2*u*_) and *wave* [*E_g_*(*y*) and *E_g_*(*x*)] modes.

## Chemical context   

Unsymmetrically *meso-*substituted porphyrins are of inter­est for a wide range of potential applications including non-linear optics (Notaras *et al.*, 2007 ▸; Zawadzka *et al.*, 2009 ▸), photodynamic therapy (Wiehe *et al.*, 2005 ▸), and sensor and device applications (Scheicher *et al.*, 2009 ▸). The synthesis of unsymmetrical porphyrin systems, such as the title compound, has been well documented (Senge *et al.*, 2010 ▸; Senge, 2011 ▸). The title compound was first synthesized as part of a study on the identification of stable porphomethenes and porphodimethenes using sterically hindered aldehydes (Senge *et al.*, 2000 ▸). This was achieved through acid-catalyzed condensation of pyrroles with aldehydes. It was later synthesized as part of this publication through the bromination of 5-*tert*-butyl­porphyrin following a reported literature procedure for similar compounds (Fazekas *et al.*, 2008 ▸) and subsequent Suzuki cross-coupling with phenyl­boronic acid, in excellent yield.
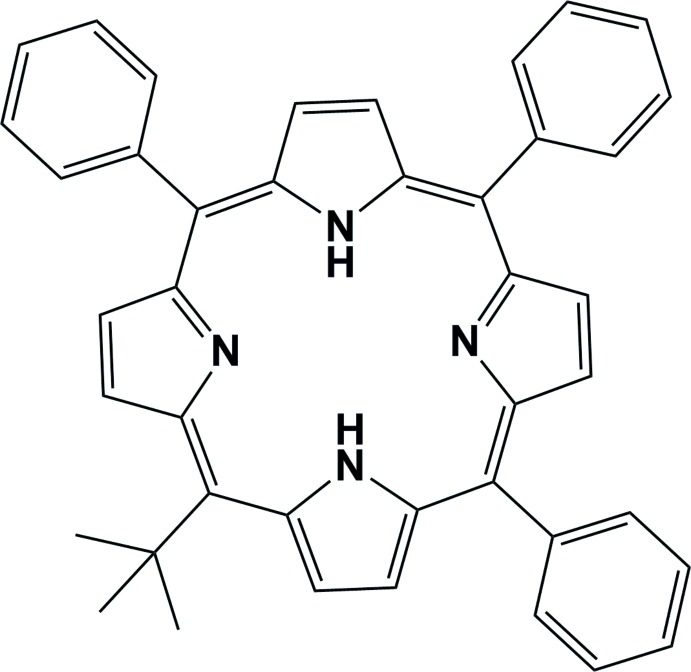


## Structural commentary   

**Figure 1:**
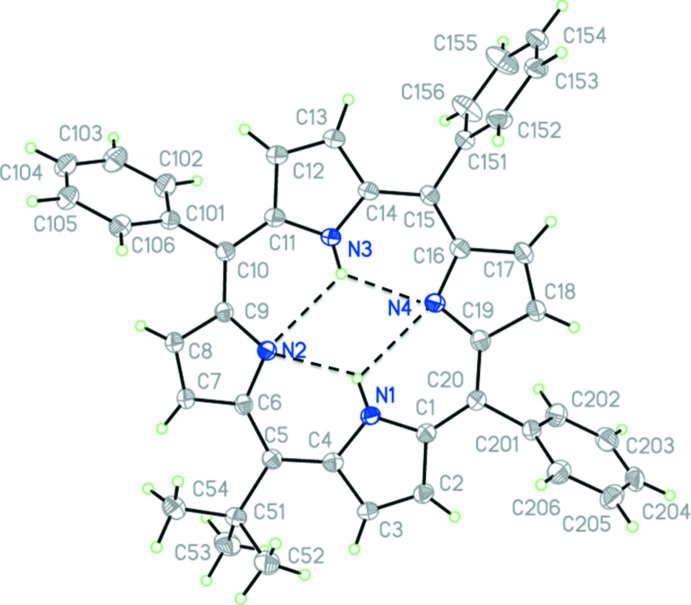
The mol­ecular structure of the title compound, with atom labelling. Displacement ellipsoids are drawn at the 50% probability level. The bifurcated N—H⋯(N,N) hydrogen bonds are shown as dashed lines (see Table 1 ▸).

The mol­ecular structure of the title compound is illustrated in Fig. 1 ▸. The distance of neighbouring N⋯N atoms in the center of the ring shows a slight elongation of the porphyrin core along direction C5 to C15 [N1⋯N2 2.818 (8) Å, N2⋯N3 2.998 (8) Å, N3⋯N4 2.830 (8) Å, N4⋯N1 2.994 (7) Å]. The NH groups are involved in intra­molecular bifurcated N—H⋯(N,N) hydrogen bonds (Table 1 ▸). The angles between the *alpha* carbons (C_a_) and the *meso* carbon atoms (C_m_) can be used to determine the structural differences between similar porphyrins and differences within the individual porphyrin structure. In the title compound, the C_a_—C_m_—C_a_ angles vary slightly with the C_a_—C_m_(*tert*-but­yl)—C_a_ angle of 120.55 (18)° at C5 representing the smallest. This is due to the nature of the *tert*-butyl substitution present. This angle is similar to that observed in the dication, 5,10,15,20-tetra­kis­(*tert*-but­yl)-22*H* ^+^,24*H* ^+^-porphyrindiium ditri­fluoro­acetate (Senge, 2000 ▸), with an average C_a_—C_m_(*tert*-but­yl)–C_a_ angle of 119.53° and 5-*tert*-butyl­porphyrin published (Ryppa *et al.*, 2005 ▸), which shows an C_a_–C_m_(*tert*-but­yl)–C_a_ angle of 119.86°. The C_a_—C_m_(phen­yl)—C_a_ angle of the title compound at C10 and C20 are quite similar at 126.03 (18) and 126.17 (18)°, respectively. The C_a_—C_m_(phen­yl)—C_a_ angles in 5,10,15,20-tetra­phenyl­porphyrin, with an average angle of 125.35° (Silvers & Tulinsky, 1967 ▸), are comparable to that of the title compound, however, the C_a_—C_m_(phen­yl)—C_a_ angle at C15 of the title compound is smaller [124.19 (18)°].

**Figure 2:**
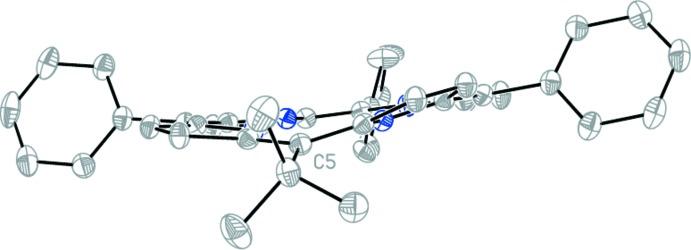
Side view of the structure of the title compound looking down the C5 *meso*-position, showing the tilt angle of the macrocycle rings. Displacement ellipsoids are drawn at the 50% probability level.

**Figure 3:**
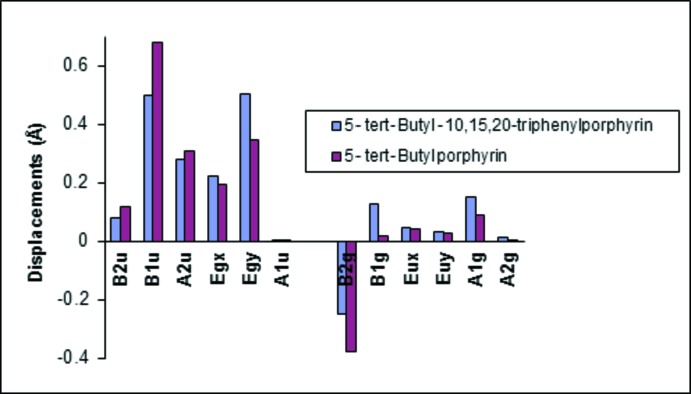
NSD analysis of the title compound and comparison with 5-*tert*-but­ylporphyrin. NSD gives a graphical representation of the displacements along the lowest frequency coordinates that best simulate the structures.

The tilt angles of the phenyl *meso*-substituents are 67.62 (2)° (C10), 71.63 (2)° (C15) and 62.42 (2)° (C20). These angles are larger than the tilt angles observed in 5,10,15,20-tetra­phenyl­porphyrin, which are *ca* 60° (Silvers & Tulinsky, 1967 ▸). The tilt of the pyrrole rings against the 24-atom plane are 9.93 (2)° (N1), 172.68 (6)° (N2), 0.17 (2)° (N3) and 3.45 (1)° (N4), with the highest deviation from the mean plane associated with the pyrrole rings closest to the *tert*-butyl group at C5. The pyrrole ring N2 shows the largest deviation and this is visible in the overall conformation of the macrocycle rings (Fig. 2 ▸). A conformational analysis (Senge *et al.*, 2015 ▸) was performed using the NSD (normal structural decomposition) method developed by Shelnutt and co-workers (Jentzen *et al.*, 1997 ▸). The conformation is characterized by a significant degree of *ruffle*d (*B*_1*u*_) distortion with contributions from *dome*d (*A*_2*u*_) and *wave* [*E_g_*(y) and *E_g_*(*x*)] modes (Fig. 3 ▸). Contributions are also evident in the *B*_2*g*_ in-plane distortion. A comparison with 5-*tert*-butylporphyrin (Ryppa *et al.*, 2005 ▸) reveals a relatively similar composition of distortion modes for both compounds. This indicates that the *tert*-butyl group is the predominant contributor to the macrocycle distortion. There is, however, a noticeable difference between the NSD of both structures with regards to the *B*_1*u*_ and *E_g_*(*y*) out-of-plane distortions. The title compound exhibits similar contributions from both these modes whereas the free base 5-*tert*-butylporphyrin shows significantly more contributions in the *B*_1*u*_ compared to the *E_g_*(*y*) distortions. This can also be seen in the in-plane distortions as both compounds show significant contributions from the *B*_2*g*_ and smaller contributions from the *A*_1*g*_ mode, the title compound shows much larger contrib­utions towards the *B*_1*g*_ in-plane distortions compared to that of the 5-*tert*-butylporphyrin.

The maximum deviations from the 24-atom mean plane are associated with carbon and nitro­gen atoms surrounding the *tert*-butyl substitution at C5. Atom C5 deviates from the mean plane by 0.440 (2) Å, whereas atoms C8, C2, C4, C20, N2 and C7 deviate from the mean plane by −0.361 (2), −0.244 (2), 0.232 (2), −0.217 (2), 0.203 (2) and −0.203 (2) Å, respectively. The smallest deviations are for the atoms associated with the pyrrole ring at the N3 position; atoms C11 C12, C13, C14 and N3 deviate from the mean plane by −0.003 (2), −0.027 (2), 0.027 (2), 0.009 (2) and −0.007 (2), respectively. This ring also shows the least tilt in the porphyrin structure.

## Supra­molecular features   

**Figure 4:**
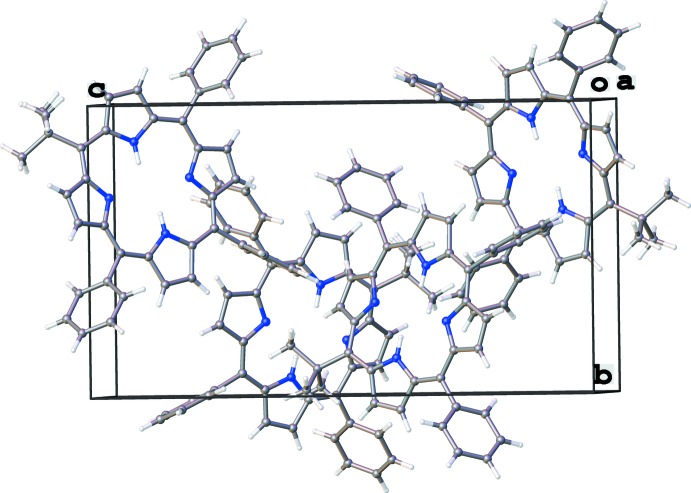
Unit cell of the title compound viewed along the *a* axis, showing four complete mol­ecular units.

**Figure 5:**
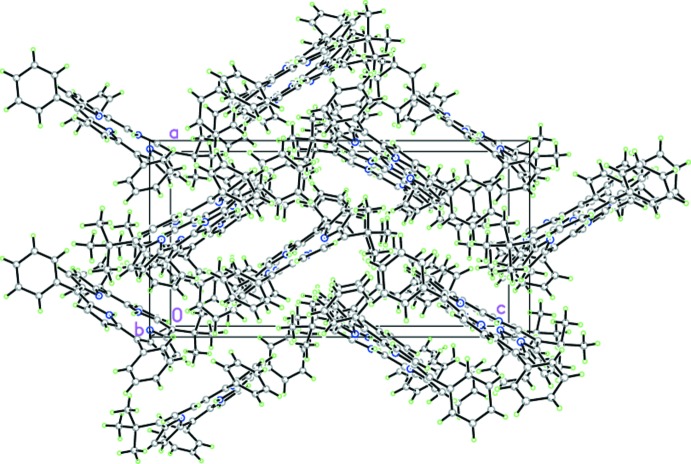
Crystal packing of the title compound, viewed along the *b* axis.

In the crystal, the four mol­ecules stack with a 90° rotation with regards to the *tert*-butyl-substituted group. The centroid–centroid distance of the 24-atom mean planes of the porphyrin rings are between 8.762 (2) and 7.758 (2) Å. The rings that stack above each other are separated by 8.762 (2) Å and the rings that are orientated in an edge-on packing are separated by a centroid–centroid distance of 7.758 (2) Å (Fig. 4 ▸). The orientation of the mol­ecules in the unit cell shows that the C_b_ *-*hydrogen atoms between the *tert*-butyl group at C5 and the phenyl group at C10 are pointing towards the center of the neighbouring ring. Mol­ecules are linked by a number of weak C—H⋯π inter­actions (Table 1 ▸), forming a three-dimensional framework. There are no solvent mol­ecules contained within the overall structure, as seen in Fig. 5 ▸.

## Database survey   

A search of the Cambridge Structural Database (CSD, Version 5.36, update November 2014; Groom & Allen, 2014 ▸) gave several hits for unsymmetrically *meso-*substituted porphyrins similar to the title compound. Speck *et al.* (1997 ▸) reported the structure of 5-(3,5-di-*tert*-butyl­muconic acid anhydride)-10,15,20-tri­phenyl­porphyrin in which they reported phenyl tilt angles of 59.07–78.15° from the 24-atom mean plane, with the largest deviation on the phenyl group opposite the alternative *meso-*substituted position. In this structure there was a larger variance of the C_a_—C_m_(phen­yl)—C_a_ angle of 123.88–125.51° and a C_a_—C_m_(C5)—C_a_ larger than the title compound of 127.35°. Senge *et al.* (1999 ▸) published the structure of 5-(2,5-di­meth­oxy­benz­yl)-10,15,20-tri­phenyl­porphyrin. The tilt angle of the phenyl rings from the 24-atom mean plane was larger and more varied compared to the title compound (73.47–87.56°). The C_a_—C_m_(phen­yl)—C_a_ angle is similar to the title compound with an angle range of 125.46–125.78°. The structure of 5-(3,5-di­hydroxy­phen­yl)-10,15,20-tri­phenyl­porphyrin pyridine clathrate has been reported by Tanaka *et al.* (2001 ▸). This compound displayed a phenyl tilt angle of 65.87–73.97° from the 24-atom mean plane and all C_a_—C_m_—C_a_ angles are of a similar size, 124.68–125.97°. Wojaczyński *et al.* (2002 ▸) reported the structure of 5,10,15-tri­phenyl­porphyrin which displays similar properties to the title compound with regards to the C_a_—C_m_(phen­yl)—C_a_ angle either side of the unsubstituted *meso* position being almost equal to each other (123.78–123.95°). The C_a_—C_m_(phen­yl)—C_a_ opposite the unsubstituted *meso* position is smaller than the C_a_—C_m_(H)—C_a_ angle, 126.20 and 127.93°, respectively. The phenyl tilt angle from the 24-atom mean plane shows a larger tilt angle (73.56–78.16°) associated with the phenyl rings. However, there is a narrower variance in these angle than in the title compound. Ryppa *et al.* (2005 ▸) published the structure of 5-*tert*-butyl­porphyrin which presents C_a_—C_m_(H)—C_a_ angles of 129.00–129.23° for the C10 and C15 positions and 125.23° for the C15 position which are all larger than in the title compound. The C_a_—C_m_(*tert*-but­yl)—C_a_ angle (C5 in both structures) are of similar size at 119.86° (120.28° for the title compound). The overall pyrrole tilt against the mean 24-atom plane shows similar results to that of the title compound. The pyrrole rings (N1 and N2) closest to the *tert*-butyl *meso* substitute show significantly higher tilts (11.68 and 14.33°, respectively) compared to the pyrrole rings (N3 and N4) closest to the unsubstituted position at C15 (4.04 and 5.26°, respectively). Yang *et al.* (2011 ▸) reported the structure ethyl 8-(10,15,20-tri­phenyl­porphyrin-5-yl)-1-naphtho­ate exhibiting a phenyl tilt angle from the 24-atom mean plane of 59.13° for the phenyl opposite the naphthanote substitute and between 74.91–76.38° for the other phenyl groups. A similar angle for all C_a_—C_m_—C_a_ is observed, 125.36–125.82°. Ma *et al.* (2013 ▸) published the structure of 2-hy­droxy­phenyl 8-(10,15,20-tri­phenyl­porphyrin-5-yl)-1-naphtho­ate which exhibited a C_a_—C_m_(phen­yl)—C_a_ angle of 124.36–124.68° similar to the title compound and a C_a_—C_m_(naphtho­ate)—C_a_ angle of 125.25° which is slightly larger compared to the title compound. The tilt angle of the phenyl rings from the 24-atom mean plane is 60.46–83.15° which shows a larger variance than for the title compound.

## Synthesis and crystallization   

The title compound was prepared previously by Senge *et al.* (2000 ▸) using a condensation approach. Here, 5-*tert*-but­ylporphyrin (100 mg, 0.27 mmol, 1 eq) was dissolved in dry CHCl_3_ (50 ml) and cooled to 273 K. *N*-Bromo­succinimide (145 mg, 0.81 mmol, 3 eq) was added and the solution was stirred for 5 h. The resulting solution was quenched with acetone and the crude product was purified *via* column chromatography on silica gel (hexa­ne/CH_2_Cl_2_ = 4:1, *v*/*v*). The solvent was removed *in vacuo* yielding 5-*tert*-but­yl-10,15,20-tri­bromo­porphyrin as purple crystals (yield: 45 mg, 0.075 mmol, 28%). *R*_f_ = 0.44 (hexa­ne:CH_2_Cl_2_, 2:1); ^1^H NMR (400 MHz, CDCl_3_) *δ*: 9.45 (*d*, *^3^J*_H-H_ = 4.76 Hz, 2H, *H_β_*), 9.36 (*d*, *^3^J*_H-H_ = 4.76 Hz, 2H, *H_β_*), 9.31 (*d*, *^3^J*_H-H_ = 5.04 Hz, 2H, *H_β_*), 9.25 (*d*, *^3^J*_H-H_ = 5 Hz, 2H, *H_β_*), 2.33 (*s*, 9H, C*H_3_*), −1.72 p.p.m. (*brs*, 2H, N*H*); HRMS (MALDI): *m*/*z* calculated for C_24_H_19_N_4_Br_3_ 600.9238 [*M* + H]^+^; found 600.9248.

A Schlenk tube was charged with 5-*tert*-but­yl-10,15,20-tri­bromo­porphyrin (20 mg, 0.033 mmol, 1 eq), phenyl­boronic acid (121.93 mg, 1 mmol, 30 eq), tetra­kis­(tri­phenyl­phosphine)palladium(0) (7.63 mg, 0.0066 mmol, 0.2 eq), cesium carbonate (651.64 mg, 2 mmol, 60 eq) and dried under vacuum. The mixture was dissolved in anhydrous THF (5 ml) and was degassed *via* three freeze–pump–thaw cycles and left under argon. The solution was heated to 353 K under an argon atmosphere for 48 h. The solvent was removed under reduced pressure and the residue was dissolved in CH_2_Cl_2_ (10 ml). The crude product was washed sequentially with sat. aq. NaHCO_3_ (20 ml) and deionized H_2_O (20 ml). The organic phase was dried over Na_2_SO_4_ and filtered. The crude product was purified *via* column chromatography on silica gel (hexa­ne/CH_2_Cl_2_ = 1:1, v/v). The solvent was removed *in vacuo*, yielding the title compound as purple crystals (yield: 15 mg, 0.025 mmol, 76%). The compound was recrystallized from CH_2_Cl_2_ layered with methanol to yield single crystals suitable for X-ray diffraction analysis.

## Refinement   

Crystal data, data collection and structure refinement details are summarized in Table 2 ▸. The structure was refined as a two-component inversion twin. The NH and C-bound H atoms were placed in their expected calculated positions and refined using a standard riding model: N—H = 0.88 Å, C—H = 0.95–0.98 Å, with *U*_iso_(H) = 1.5*U*_eq_(C-meth­yl) and 1.2*U*_eq_(N,C) for other H atoms.

## Supplementary Material

Crystal structure: contains datablock(s) I, Global. DOI: 10.1107/S2056989016000025/su5266sup1.cif

Structure factors: contains datablock(s) I. DOI: 10.1107/S2056989016000025/su5266Isup2.hkl

CCDC reference: 1444998

Additional supporting information:  crystallographic information; 3D view; checkCIF report

## Figures and Tables

**Table 1:** Hydrogen-bond geometry (Å, °) *Cg*1, *Cg*2, *Cg*3, *Cg*4 and *Cg*6 are the centroids of rings N1/C1–C4, N2/C6–C9, N3/C11–C14, N4/C16–C19 and C151–C156, respectively.

*D*—H⋯*A*	*D*—H	H⋯*A*	*D*⋯*A*	*D*—H⋯*A*
N1—H1*A*⋯N2	0.88	2.24	2.818 (2)	123
N1—H1*A*⋯N4	0.88	2.46	2.995 (2)	119
N3—H3*A*⋯N2	0.88	2.46	2.998 (2)	120
N3—H3*A*⋯N4	0.88	2.26	2.831 (2)	123
C204—H1⋯*Cg*6^i^	0.95	2.57	3.477 (3)	160
C202—H4⋯*Cg*3^ii^	0.95	2.61	3.511 (2)	160
C8—H8⋯*Cg*1^iii^	0.95	2.67	3.452 (2)	140
C156—H18⋯*Cg*1^iv^	0.95	2.90	3.610 (2)	132
C154—H20⋯*Cg*2^v^	0.95	2.78	3.654 (2)	153
C54—H30⋯*Cg*4^iii^	0.98	2.98	3.664 (2)	128

Symmetry codes: (i) 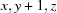; (ii) 
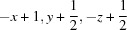
; (iii) 
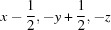
; (iv) 
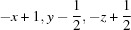
; (v) 
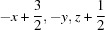
.

**Table 2:** Experimental details

Crystal data	
Chemical formula	C_42_H_34_N_4_
*M*_r_	594.73
Crystal system, space group	Orthorhombic, *P*2_1_2_1_2_1_
Temperature (K)	100
*a*, *b*, *c* (Å)	11.3373 (4), 12.6936 (5), 21.9616 (8)
*V* (Å^3^)	3160.5 (2)
*Z*	4
Radiation type	Mo *K*α
μ (mm^−1^)	0.07
Crystal size (mm)	0.34 × 0.30 × 0.30

Data collection	
Diffractometer	Bruker SMART APEXII
Absorption correction	Multi-scan (*SADABS*; Bruker, 2014 ▸)
*T*_min_, *T*_max_	0.697, 0.746
No. of measured, independent and observed [*I* > 2σ(*I*)] reflections	124779, 7377, 7039
*R*_int_	0.029
(sin θ/λ)_max_ (Å^−1^)	0.653

Refinement	
*R*[*F* ^2^ > 2σ(*F* ^2^)], *wR*(*F* ^2^), *S*	0.038, 0.106, 1.06
No. of reflections	7377
No. of parameters	419
H-atom treatment	H-atom parameters constrained
Δρ_max_, Δρ_min_ (e Å^−3^)	0.30, −0.22
Absolute structure	Refined as an inversion twin

Computer programs: *APEX2* and *SAINT-Plus* (Bruker, 2014 ▸), *SHELXT* (Sheldrick, 2015*a* ▸), *SHELXL2014/7* (Sheldrick, 2015*b* ▸), *XP* in *SHELXTL* (Sheldrick, 2008 ▸) and *publCIF* (Westrip, 2010 ▸).

## supplementary crystallographic information

### Crystal data

**Table:**

C_42_H_34_N_4_	*D*_x_ = 1.250 Mg m^−^^3^
*M**_r_* = 594.73	Mo *K*α radiation, λ = 0.71073 Å
Orthorhombic, *P*2_1_2_1_2_1_	Cell parameters from 9394 reflections
*a* = 11.3373 (4) Å	θ = 2.4–27.6°
*b* = 12.6936 (5) Å	µ = 0.07 mm^−^^1^
*c* = 21.9616 (8) Å	*T* = 100 K
*V* = 3160.5 (2) Å^3^	Block, purple
*Z* = 4	0.34 × 0.30 × 0.30 mm
*F*(000) = 1256	

### Data collection

**Table:**

Bruker SMART APEX2 area detector diffractometer	7377 independent reflections
Radiation source: sealed tube	7039 reflections with *I* > 2σ(*I*)
Detector resolution: 8.258 pixels mm^-1^	*R*_int_ = 0.029
φ and ω scans	θ_max_ = 27.7°, θ_min_ = 1.9°
Absorption correction: multi-scan (*SADABS*; Bruker, 2014)	*h* = −14→14
*T*_min_ = 0.697, *T*_max_ = 0.746	*k* = −16→16
124779 measured reflections	*l* = −28→28

### Refinement

**Table:**

Refinement on *F*^2^	Hydrogen site location: inferred from neighbouring sites
Least-squares matrix: full	H-atom parameters constrained
*R*[*F*^2^ > 2σ(*F*^2^)] = 0.038	*w* = 1/[σ^2^(*F*_o_^2^) + (0.0624*P*)^2^ + 0.8289*P*] where *P* = (*F*_o_^2^ + 2*F*_c_^2^)/3
*wR*(*F*^2^) = 0.106	(Δ/σ)_max_ < 0.001
*S* = 1.06	Δρ_max_ = 0.30 e Å^−^^3^
7377 reflections	Δρ_min_ = −0.22 e Å^−^^3^
419 parameters	Absolute structure: Refined as an inversion twin
0 restraints	

### Special details

**Table:**

Geometry. All e.s.d.'s (except the e.s.d. in the dihedral angle between two l.s. planes) are estimated using the full covariance matrix. The cell e.s.d.'s are taken into account individually in the estimation of e.s.d.'s in distances, angles and torsion angles; correlations between e.s.d.'s in cell parameters are only used when they are defined by crystal symmetry. An approximate (isotropic) treatment of cell e.s.d.'s is used for estimating e.s.d.'s involving l.s. planes.
Refinement. Refined as a 2-component inversion twin.

### Fractional atomic coordinates and isotropic or equivalent isotropic displacement parameters (Å^2^)

**Table:**

	*x*	*y*	*z*	*U*_iso_*/*U*_eq_	
C1	0.62591 (17)	0.45488 (15)	0.12493 (9)	0.0203 (4)	
C2	0.59743 (18)	0.55211 (16)	0.09532 (9)	0.0229 (4)	
H32	0.6114	0.6207	0.1110	0.027*	
C3	0.54693 (19)	0.52918 (16)	0.04083 (10)	0.0239 (4)	
H33	0.5204	0.5793	0.0117	0.029*	
C4	0.54015 (17)	0.41647 (15)	0.03442 (9)	0.0204 (4)	
C5	0.48857 (17)	0.35700 (16)	−0.01260 (9)	0.0209 (4)	
C6	0.45205 (17)	0.25105 (16)	−0.00211 (9)	0.0196 (4)	
C7	0.35626 (19)	0.19968 (16)	−0.03491 (9)	0.0227 (4)	
H7	0.3092	0.2299	−0.0662	0.027*	
C8	0.34703 (18)	0.10171 (16)	−0.01265 (9)	0.0228 (4)	
H8	0.2923	0.0493	−0.0251	0.027*	
C9	0.43661 (17)	0.09110 (15)	0.03413 (9)	0.0197 (4)	
C10	0.45654 (18)	−0.00410 (16)	0.06508 (9)	0.0212 (4)	
C11	0.52777 (18)	−0.01752 (16)	0.11669 (9)	0.0229 (4)	
C12	0.5508 (2)	−0.11397 (17)	0.14810 (10)	0.0289 (5)	
H14	0.5230	−0.1817	0.1366	0.035*	
C13	0.6196 (2)	−0.09202 (17)	0.19738 (11)	0.0286 (5)	
H15	0.6477	−0.1415	0.2264	0.034*	
C14	0.64198 (18)	0.01871 (16)	0.19762 (9)	0.0220 (4)	
C15	0.71165 (17)	0.07601 (16)	0.23872 (9)	0.0213 (4)	
C16	0.73131 (18)	0.18453 (16)	0.23489 (9)	0.0220 (4)	
C17	0.8031 (2)	0.24329 (18)	0.27767 (9)	0.0269 (4)	
H17	0.8470	0.2152	0.3108	0.032*	
C18	0.7955 (2)	0.34552 (17)	0.26138 (10)	0.0265 (4)	
H16	0.8332	0.4034	0.2807	0.032*	
C19	0.71826 (18)	0.34990 (17)	0.20853 (9)	0.0222 (4)	
C20	0.68693 (17)	0.44495 (16)	0.17978 (9)	0.0207 (4)	
C51	0.4682 (2)	0.40954 (17)	−0.07592 (10)	0.0258 (4)	
C52	0.5728 (3)	0.4822 (2)	−0.09285 (11)	0.0416 (6)	
H26	0.5619	0.5091	−0.1343	0.062*	
H28	0.6466	0.4422	−0.0907	0.062*	
H27	0.5762	0.5415	−0.0643	0.062*	
C53	0.3523 (2)	0.4725 (2)	−0.07555 (12)	0.0379 (6)	
H25	0.3423	0.5081	−0.1148	0.057*	
H23	0.3547	0.5251	−0.0429	0.057*	
H24	0.2859	0.4245	−0.0687	0.057*	
C54	0.4670 (2)	0.32888 (19)	−0.12892 (10)	0.0328 (5)	
H30	0.3922	0.2902	−0.1286	0.049*	
H29	0.5325	0.2793	−0.1240	0.049*	
H31	0.4754	0.3662	−0.1677	0.049*	
C101	0.39426 (18)	−0.10025 (16)	0.04186 (10)	0.0237 (4)	
C102	0.3099 (2)	−0.15159 (18)	0.07717 (11)	0.0309 (5)	
H11	0.2940	−0.1273	0.1173	0.037*	
C103	0.2487 (2)	−0.2378 (2)	0.05445 (12)	0.0376 (5)	
H12	0.1914	−0.2718	0.0792	0.045*	
C104	0.2698 (2)	−0.27463 (18)	−0.00350 (12)	0.0355 (5)	
H2	0.2257	−0.3321	−0.0194	0.043*	
C105	0.3566 (2)	−0.22658 (19)	−0.03835 (12)	0.0349 (5)	
H10	0.3742	−0.2533	−0.0777	0.042*	
C106	0.4181 (2)	−0.13967 (18)	−0.01629 (11)	0.0305 (5)	
H9	0.4765	−0.1070	−0.0409	0.037*	
C151	0.76452 (18)	0.01846 (16)	0.29167 (9)	0.0224 (4)	
C152	0.8858 (2)	0.00258 (18)	0.29672 (11)	0.0296 (5)	
H22	0.9363	0.0245	0.2646	0.035*	
C153	0.9336 (2)	−0.04490 (17)	0.34815 (11)	0.0308 (5)	
H21	1.0165	−0.0550	0.3509	0.037*	
C154	0.8620 (2)	−0.07733 (18)	0.39504 (10)	0.0303 (5)	
H20	0.8952	−0.1086	0.4304	0.036*	
C155	0.7411 (2)	−0.0641 (3)	0.39028 (11)	0.0439 (7)	
H19	0.6910	−0.0871	0.4223	0.053*	
C156	0.6928 (2)	−0.0172 (2)	0.33863 (10)	0.0376 (6)	
H18	0.6097	−0.0095	0.3355	0.045*	
C201	0.72236 (19)	0.54413 (16)	0.21163 (10)	0.0240 (4)	
C202	0.6685 (2)	0.56983 (18)	0.26648 (10)	0.0315 (5)	
H4	0.6079	0.5260	0.2823	0.038*	
C203	0.7030 (3)	0.65957 (19)	0.29837 (11)	0.0376 (6)	
H5	0.6658	0.6771	0.3358	0.045*	
C204	0.7912 (3)	0.72280 (18)	0.27563 (12)	0.0394 (6)	
H1	0.8155	0.7834	0.2977	0.047*	
C205	0.8445 (2)	0.69838 (19)	0.22084 (13)	0.0379 (6)	
H34	0.9050	0.7425	0.2052	0.045*	
C206	0.8097 (2)	0.60957 (18)	0.18865 (11)	0.0309 (5)	
H3	0.8459	0.5935	0.1507	0.037*	
N1	0.58622 (15)	0.37541 (13)	0.08755 (7)	0.0195 (3)	
H1A	0.5897	0.3078	0.0963	0.023*	
N2	0.49590 (15)	0.18454 (13)	0.04146 (8)	0.0194 (3)	
N3	0.58505 (15)	0.06048 (13)	0.14814 (7)	0.0206 (3)	
H3A	0.5852	0.1276	0.1380	0.025*	
N4	0.68278 (15)	0.25104 (13)	0.19218 (7)	0.0207 (3)	

### Atomic displacement parameters (Å^2^)

**Table:**

	*U*^11^	*U*^22^	*U*^33^	*U*^12^	*U*^13^	*U*^23^
C1	0.0194 (8)	0.0195 (9)	0.0221 (9)	0.0008 (7)	0.0007 (7)	−0.0005 (7)
C2	0.0248 (9)	0.0176 (9)	0.0263 (9)	0.0017 (7)	−0.0015 (8)	−0.0013 (7)
C3	0.0287 (10)	0.0171 (9)	0.0260 (9)	0.0009 (8)	−0.0027 (8)	0.0024 (7)
C4	0.0178 (8)	0.0203 (9)	0.0230 (9)	0.0019 (7)	0.0005 (7)	0.0012 (7)
C5	0.0185 (8)	0.0215 (9)	0.0227 (9)	0.0018 (7)	−0.0010 (7)	0.0005 (7)
C6	0.0193 (8)	0.0212 (9)	0.0183 (8)	0.0015 (7)	0.0002 (7)	−0.0026 (7)
C7	0.0239 (9)	0.0227 (9)	0.0214 (9)	−0.0014 (7)	−0.0042 (8)	−0.0013 (7)
C8	0.0234 (9)	0.0223 (9)	0.0228 (9)	−0.0011 (7)	−0.0041 (8)	−0.0009 (8)
C9	0.0197 (9)	0.0208 (9)	0.0186 (9)	0.0004 (7)	0.0001 (7)	−0.0011 (7)
C10	0.0223 (9)	0.0204 (9)	0.0208 (9)	0.0013 (7)	−0.0012 (7)	−0.0007 (7)
C11	0.0237 (9)	0.0198 (9)	0.0251 (9)	0.0021 (7)	−0.0027 (8)	0.0010 (8)
C12	0.0348 (11)	0.0200 (10)	0.0320 (11)	0.0012 (9)	−0.0100 (9)	0.0025 (8)
C13	0.0336 (11)	0.0219 (9)	0.0305 (11)	0.0002 (9)	−0.0091 (9)	0.0049 (8)
C14	0.0235 (9)	0.0204 (9)	0.0221 (9)	0.0015 (7)	−0.0027 (7)	0.0036 (7)
C15	0.0199 (9)	0.0232 (9)	0.0207 (9)	0.0024 (7)	−0.0021 (7)	0.0013 (7)
C16	0.0228 (9)	0.0232 (9)	0.0199 (9)	0.0017 (7)	−0.0017 (7)	0.0016 (7)
C17	0.0303 (10)	0.0283 (10)	0.0220 (10)	0.0021 (9)	−0.0087 (8)	0.0002 (8)
C18	0.0316 (11)	0.0231 (10)	0.0248 (10)	0.0011 (8)	−0.0081 (9)	−0.0020 (8)
C19	0.0208 (9)	0.0247 (10)	0.0213 (9)	0.0005 (8)	−0.0002 (8)	−0.0011 (8)
C20	0.0201 (9)	0.0204 (9)	0.0215 (9)	−0.0004 (7)	0.0011 (7)	−0.0008 (7)
C51	0.0293 (10)	0.0221 (9)	0.0260 (10)	−0.0005 (8)	−0.0052 (8)	0.0042 (8)
C52	0.0548 (16)	0.0427 (14)	0.0274 (11)	−0.0190 (13)	−0.0019 (11)	0.0082 (10)
C53	0.0417 (13)	0.0336 (12)	0.0385 (12)	0.0135 (11)	−0.0096 (11)	0.0046 (10)
C54	0.0441 (13)	0.0330 (12)	0.0213 (10)	−0.0018 (10)	0.0011 (9)	0.0016 (8)
C101	0.0259 (10)	0.0196 (9)	0.0255 (10)	0.0028 (8)	−0.0049 (8)	0.0002 (8)
C102	0.0344 (11)	0.0281 (11)	0.0302 (11)	−0.0039 (9)	0.0025 (9)	−0.0032 (9)
C103	0.0380 (12)	0.0307 (12)	0.0443 (14)	−0.0073 (10)	0.0028 (10)	−0.0021 (10)
C104	0.0408 (13)	0.0218 (10)	0.0440 (13)	−0.0002 (9)	−0.0103 (11)	−0.0040 (9)
C105	0.0476 (14)	0.0243 (10)	0.0328 (12)	0.0053 (10)	−0.0051 (11)	−0.0071 (9)
C106	0.0402 (12)	0.0233 (10)	0.0279 (10)	0.0027 (9)	−0.0014 (9)	−0.0017 (8)
C151	0.0254 (10)	0.0206 (9)	0.0212 (9)	0.0004 (8)	−0.0042 (7)	0.0010 (7)
C152	0.0256 (10)	0.0269 (10)	0.0362 (11)	−0.0030 (8)	−0.0019 (9)	0.0117 (9)
C153	0.0260 (10)	0.0250 (10)	0.0414 (12)	−0.0022 (9)	−0.0108 (9)	0.0083 (9)
C154	0.0394 (12)	0.0274 (11)	0.0242 (10)	0.0065 (9)	−0.0096 (9)	0.0011 (8)
C155	0.0403 (13)	0.0683 (19)	0.0231 (11)	0.0171 (13)	0.0068 (10)	0.0140 (11)
C156	0.0275 (11)	0.0577 (16)	0.0277 (11)	0.0117 (11)	0.0035 (9)	0.0128 (11)
C201	0.0256 (9)	0.0213 (9)	0.0251 (9)	0.0004 (8)	−0.0058 (8)	−0.0008 (8)
C202	0.0390 (12)	0.0272 (11)	0.0283 (11)	0.0000 (9)	−0.0015 (9)	−0.0046 (9)
C203	0.0562 (16)	0.0270 (11)	0.0297 (11)	0.0092 (11)	−0.0103 (11)	−0.0074 (9)
C204	0.0527 (15)	0.0217 (10)	0.0439 (14)	0.0055 (10)	−0.0272 (12)	−0.0059 (9)
C205	0.0335 (12)	0.0240 (11)	0.0561 (16)	−0.0016 (9)	−0.0128 (11)	0.0019 (10)
C206	0.0262 (10)	0.0271 (11)	0.0395 (12)	0.0008 (9)	−0.0024 (9)	0.0005 (9)
N1	0.0204 (7)	0.0165 (7)	0.0217 (8)	0.0001 (6)	−0.0010 (6)	0.0015 (6)
N2	0.0210 (8)	0.0185 (8)	0.0186 (7)	0.0009 (6)	−0.0001 (6)	−0.0004 (6)
N3	0.0232 (8)	0.0174 (7)	0.0213 (8)	0.0004 (6)	−0.0032 (6)	0.0032 (6)
N4	0.0225 (8)	0.0199 (8)	0.0197 (8)	0.0007 (7)	−0.0008 (6)	0.0024 (6)

### Geometric parameters (Å, º)

**Table:**

C1—N1	1.376 (2)	C52—H28	0.9800
C1—C20	1.395 (3)	C52—H27	0.9800
C1—C2	1.432 (3)	C53—H25	0.9800
C2—C3	1.358 (3)	C53—H23	0.9800
C2—H32	0.9500	C53—H24	0.9800
C3—C4	1.440 (3)	C54—H30	0.9800
C3—H33	0.9500	C54—H29	0.9800
C4—N1	1.381 (2)	C54—H31	0.9800
C4—C5	1.407 (3)	C101—C102	1.393 (3)
C5—C6	1.426 (3)	C101—C106	1.398 (3)
C5—C51	1.559 (3)	C102—C103	1.388 (3)
C6—N2	1.369 (3)	C102—H11	0.9500
C6—C7	1.457 (3)	C103—C104	1.377 (4)
C7—C8	1.340 (3)	C103—H12	0.9500
C7—H7	0.9500	C104—C105	1.387 (4)
C8—C9	1.451 (3)	C104—H2	0.9500
C8—H8	0.9500	C105—C106	1.392 (3)
C9—N2	1.373 (2)	C105—H10	0.9500
C9—C10	1.405 (3)	C106—H9	0.9500
C10—C11	1.402 (3)	C151—C156	1.389 (3)
C10—C101	1.499 (3)	C151—C152	1.394 (3)
C11—N3	1.371 (3)	C152—C153	1.390 (3)
C11—C12	1.429 (3)	C152—H22	0.9500
C12—C13	1.363 (3)	C153—C154	1.374 (3)
C12—H14	0.9500	C153—H21	0.9500
C13—C14	1.428 (3)	C154—C155	1.385 (4)
C13—H15	0.9500	C154—H20	0.9500
C14—N3	1.371 (2)	C155—C156	1.393 (3)
C14—C15	1.403 (3)	C155—H19	0.9500
C15—C16	1.398 (3)	C156—H18	0.9500
C15—C151	1.499 (3)	C201—C206	1.388 (3)
C16—N4	1.377 (2)	C201—C202	1.389 (3)
C16—C17	1.450 (3)	C202—C203	1.393 (3)
C17—C18	1.349 (3)	C202—H4	0.9500
C17—H17	0.9500	C203—C204	1.376 (4)
C18—C19	1.455 (3)	C203—H5	0.9500
C18—H16	0.9500	C204—C205	1.382 (4)
C19—N4	1.366 (3)	C204—H1	0.9500
C19—C20	1.407 (3)	C205—C206	1.388 (3)
C20—C201	1.495 (3)	C205—H34	0.9500
C51—C53	1.539 (3)	C206—H3	0.9500
C51—C52	1.548 (3)	N1—H1A	0.8800
C51—C54	1.550 (3)	N3—H3A	0.8800
C52—H26	0.9800		
			
N1—C1—C20	127.66 (18)	C51—C53—H23	109.5
N1—C1—C2	106.69 (16)	H25—C53—H23	109.5
C20—C1—C2	125.59 (18)	C51—C53—H24	109.5
C3—C2—C1	108.08 (18)	H25—C53—H24	109.5
C3—C2—H32	126.0	H23—C53—H24	109.5
C1—C2—H32	126.0	C51—C54—H30	109.5
C2—C3—C4	108.77 (18)	C51—C54—H29	109.5
C2—C3—H33	125.6	H30—C54—H29	109.5
C4—C3—H33	125.6	C51—C54—H31	109.5
N1—C4—C5	125.11 (17)	H30—C54—H31	109.5
N1—C4—C3	105.80 (17)	H29—C54—H31	109.5
C5—C4—C3	128.86 (19)	C102—C101—C106	118.3 (2)
C4—C5—C6	120.55 (18)	C102—C101—C10	120.98 (19)
C4—C5—C51	119.12 (18)	C106—C101—C10	120.7 (2)
C6—C5—C51	120.31 (17)	C103—C102—C101	120.8 (2)
N2—C6—C5	126.09 (17)	C103—C102—H11	119.6
N2—C6—C7	109.88 (17)	C101—C102—H11	119.6
C5—C6—C7	123.95 (18)	C104—C103—C102	120.9 (2)
C8—C7—C6	107.04 (18)	C104—C103—H12	119.6
C8—C7—H7	126.5	C102—C103—H12	119.6
C6—C7—H7	126.5	C103—C104—C105	118.9 (2)
C7—C8—C9	106.84 (18)	C103—C104—H2	120.5
C7—C8—H8	126.6	C105—C104—H2	120.5
C9—C8—H8	126.6	C104—C105—C106	120.8 (2)
N2—C9—C10	127.43 (17)	C104—C105—H10	119.6
N2—C9—C8	110.22 (17)	C106—C105—H10	119.6
C10—C9—C8	122.34 (18)	C105—C106—C101	120.3 (2)
C11—C10—C9	126.03 (18)	C105—C106—H9	119.8
C11—C10—C101	116.57 (18)	C101—C106—H9	119.8
C9—C10—C101	117.38 (17)	C156—C151—C152	118.1 (2)
N3—C11—C10	126.33 (18)	C156—C151—C15	120.06 (19)
N3—C11—C12	106.79 (17)	C152—C151—C15	121.78 (19)
C10—C11—C12	126.84 (19)	C153—C152—C151	120.8 (2)
C13—C12—C11	108.22 (19)	C153—C152—H22	119.6
C13—C12—H14	125.9	C151—C152—H22	119.6
C11—C12—H14	125.9	C154—C153—C152	120.5 (2)
C12—C13—C14	107.81 (19)	C154—C153—H21	119.7
C12—C13—H15	126.1	C152—C153—H21	119.7
C14—C13—H15	126.1	C153—C154—C155	119.5 (2)
N3—C14—C15	125.08 (18)	C153—C154—H20	120.3
N3—C14—C13	107.10 (18)	C155—C154—H20	120.3
C15—C14—C13	127.78 (19)	C154—C155—C156	120.1 (2)
C16—C15—C14	124.19 (18)	C154—C155—H19	119.9
C16—C15—C151	117.60 (18)	C156—C155—H19	119.9
C14—C15—C151	118.16 (18)	C151—C156—C155	120.9 (2)
N4—C16—C15	125.56 (18)	C151—C156—H18	119.5
N4—C16—C17	110.51 (18)	C155—C156—H18	119.5
C15—C16—C17	123.88 (18)	C206—C201—C202	119.2 (2)
C18—C17—C16	106.69 (18)	C206—C201—C20	121.71 (19)
C18—C17—H17	126.7	C202—C201—C20	119.03 (19)
C16—C17—H17	126.7	C201—C202—C203	120.3 (2)
C17—C18—C19	106.67 (19)	C201—C202—H4	119.9
C17—C18—H16	126.7	C203—C202—H4	119.9
C19—C18—H16	126.7	C204—C203—C202	119.9 (2)
N4—C19—C20	126.54 (18)	C204—C203—H5	120.0
N4—C19—C18	110.60 (18)	C202—C203—H5	120.0
C20—C19—C18	122.86 (19)	C203—C204—C205	120.2 (2)
C1—C20—C19	126.17 (18)	C203—C204—H1	119.9
C1—C20—C201	117.46 (18)	C205—C204—H1	119.9
C19—C20—C201	116.37 (17)	C204—C205—C206	120.1 (2)
C53—C51—C52	110.2 (2)	C204—C205—H34	119.9
C53—C51—C54	109.82 (19)	C206—C205—H34	119.9
C52—C51—C54	102.74 (19)	C205—C206—C201	120.2 (2)
C53—C51—C5	110.11 (18)	C205—C206—H3	119.9
C52—C51—C5	110.86 (18)	C201—C206—H3	119.9
C54—C51—C5	112.86 (17)	C1—N1—C4	110.55 (16)
C51—C52—H26	109.5	C1—N1—H1A	124.7
C51—C52—H28	109.5	C4—N1—H1A	124.7
H26—C52—H28	109.5	C6—N2—C9	105.85 (16)
C51—C52—H27	109.5	C14—N3—C11	110.07 (16)
H26—C52—H27	109.5	C14—N3—H3A	125.0
H28—C52—H27	109.5	C11—N3—H3A	125.0
C51—C53—H25	109.5	C19—N4—C16	105.46 (17)
			
N1—C1—C2—C3	−2.5 (2)	C6—C5—C51—C52	−142.9 (2)
C20—C1—C2—C3	174.9 (2)	C4—C5—C51—C54	153.5 (2)
C1—C2—C3—C4	0.8 (2)	C6—C5—C51—C54	−28.3 (3)
C2—C3—C4—N1	1.2 (2)	C11—C10—C101—C102	63.5 (3)
C2—C3—C4—C5	175.9 (2)	C9—C10—C101—C102	−115.2 (2)
N1—C4—C5—C6	16.6 (3)	C11—C10—C101—C106	−117.5 (2)
C3—C4—C5—C6	−157.2 (2)	C9—C10—C101—C106	63.7 (3)
N1—C4—C5—C51	−165.28 (18)	C106—C101—C102—C103	−1.8 (3)
C3—C4—C5—C51	21.0 (3)	C10—C101—C102—C103	177.1 (2)
C4—C5—C6—N2	−24.7 (3)	C101—C102—C103—C104	0.0 (4)
C51—C5—C6—N2	157.20 (19)	C102—C103—C104—C105	2.3 (4)
C4—C5—C6—C7	151.72 (19)	C103—C104—C105—C106	−2.7 (4)
C51—C5—C6—C7	−26.4 (3)	C104—C105—C106—C101	0.8 (4)
N2—C6—C7—C8	−2.7 (2)	C102—C101—C106—C105	1.4 (3)
C5—C6—C7—C8	−179.56 (19)	C10—C101—C106—C105	−177.5 (2)
C6—C7—C8—C9	0.0 (2)	C16—C15—C151—C156	107.2 (2)
C7—C8—C9—N2	2.6 (2)	C14—C15—C151—C156	−70.3 (3)
C7—C8—C9—C10	−176.48 (19)	C16—C15—C151—C152	−70.3 (3)
N2—C9—C10—C11	11.5 (3)	C14—C15—C151—C152	112.2 (2)
C8—C9—C10—C11	−169.67 (19)	C156—C151—C152—C153	−1.8 (4)
N2—C9—C10—C101	−169.90 (19)	C15—C151—C152—C153	175.7 (2)
C8—C9—C10—C101	9.0 (3)	C151—C152—C153—C154	0.1 (4)
C9—C10—C11—N3	4.0 (3)	C152—C153—C154—C155	1.2 (4)
C101—C10—C11—N3	−174.65 (19)	C153—C154—C155—C156	−0.8 (4)
C9—C10—C11—C12	−178.7 (2)	C152—C151—C156—C155	2.2 (4)
C101—C10—C11—C12	2.6 (3)	C15—C151—C156—C155	−175.4 (3)
N3—C11—C12—C13	0.6 (3)	C154—C155—C156—C151	−1.0 (5)
C10—C11—C12—C13	−177.1 (2)	C1—C20—C201—C206	−70.1 (3)
C11—C12—C13—C14	−0.5 (3)	C19—C20—C201—C206	110.2 (2)
C12—C13—C14—N3	0.2 (3)	C1—C20—C201—C202	111.6 (2)
C12—C13—C14—C15	−177.8 (2)	C19—C20—C201—C202	−68.1 (3)
N3—C14—C15—C16	0.7 (3)	C206—C201—C202—C203	−0.8 (3)
C13—C14—C15—C16	178.4 (2)	C20—C201—C202—C203	177.5 (2)
N3—C14—C15—C151	178.04 (19)	C201—C202—C203—C204	−0.3 (4)
C13—C14—C15—C151	−4.2 (3)	C202—C203—C204—C205	0.9 (4)
C14—C15—C16—N4	2.4 (3)	C203—C204—C205—C206	−0.3 (4)
C151—C15—C16—N4	−174.98 (19)	C204—C205—C206—C201	−0.8 (4)
C14—C15—C16—C17	179.8 (2)	C202—C201—C206—C205	1.4 (3)
C151—C15—C16—C17	2.4 (3)	C20—C201—C206—C205	−176.9 (2)
N4—C16—C17—C18	1.3 (3)	C20—C1—N1—C4	−174.01 (19)
C15—C16—C17—C18	−176.4 (2)	C2—C1—N1—C4	3.3 (2)
C16—C17—C18—C19	0.3 (2)	C5—C4—N1—C1	−177.77 (18)
C17—C18—C19—N4	−1.8 (3)	C3—C4—N1—C1	−2.8 (2)
C17—C18—C19—C20	178.2 (2)	C5—C6—N2—C9	−179.02 (19)
N1—C1—C20—C19	−1.3 (3)	C7—C6—N2—C9	4.1 (2)
C2—C1—C20—C19	−178.2 (2)	C10—C9—N2—C6	174.85 (19)
N1—C1—C20—C201	179.02 (19)	C8—C9—N2—C6	−4.1 (2)
C2—C1—C20—C201	2.2 (3)	C15—C14—N3—C11	178.23 (19)
N4—C19—C20—C1	−8.5 (3)	C13—C14—N3—C11	0.1 (2)
C18—C19—C20—C1	171.5 (2)	C10—C11—N3—C14	177.3 (2)
N4—C19—C20—C201	171.15 (19)	C12—C11—N3—C14	−0.4 (2)
C18—C19—C20—C201	−8.8 (3)	C20—C19—N4—C16	−177.4 (2)
C4—C5—C51—C53	−83.4 (2)	C18—C19—N4—C16	2.6 (2)
C6—C5—C51—C53	94.8 (2)	C15—C16—N4—C19	175.3 (2)
C4—C5—C51—C52	38.9 (3)	C17—C16—N4—C19	−2.4 (2)

### Hydrogen-bond geometry (Å, º)

Cg1, Cg2, Cg3, Cg4 and Cg6 are the centroids of rings N1/C1–C4, N2/C6–C9, N3/C11–C14, N4/C16–C19 and C151–C156, respectively.

**Table:**

*D*—H···*A*	*D*—H	H···*A*	*D*···*A*	*D*—H···*A*
N1—H1*A*···N2	0.88	2.24	2.818 (2)	123
N1—H1*A*···N4	0.88	2.46	2.995 (2)	119
N3—H3*A*···N2	0.88	2.46	2.998 (2)	120
N3—H3*A*···N4	0.88	2.26	2.831 (2)	123
C204—H1···*Cg*6^i^	0.95	2.57	3.477 (3)	160
C202—H4···*Cg*3^ii^	0.95	2.61	3.511 (2)	160
C8—H8···*Cg*1^iii^	0.95	2.67	3.452 (2)	140
C156—H18···*Cg*1^iv^	0.95	2.90	3.610 (2)	132
C154—H20···*Cg*2^v^	0.95	2.78	3.654 (2)	153
C54—H30···*Cg*4^iii^	0.98	2.98	3.664 (2)	128

Symmetry codes: (i) *x*, *y*+1, *z*; (ii) −*x*+1, *y*+1/2, −*z*+1/2; (iii) *x*−1/2, −*y*+1/2, −*z*; (iv) −*x*+1, *y*−1/2, −*z*+1/2; (v) −*x*+3/2, −*y*, *z*+1/2.
